# Effects of increase in fish oil intake on intestinal eicosanoids and inflammation in a mouse model of colitis

**DOI:** 10.1186/1476-511X-12-81

**Published:** 2013-05-31

**Authors:** Nabil Bosco, Viral Brahmbhatt, Manuel Oliveira, Francois-Pierre Martin, Pia Lichti, Frederic Raymond, Robert Mansourian, Sylviane Metairon, Cecil Pace-Asciak, Viktoria Bastic Schmid, Serge Rezzi, Dirk Haller, Jalil Benyacoub

**Affiliations:** 1 Nestlé Research Center, Vers-chez-les-Blanc, Lausanne 26, CH-1000, Switzerland; 2 Technische Universität München, Biofunctionality, ZIEL–Research Center for Nutrition and Food Science, CDD - Center for Diet and Disease, Gregor-Mendel-Straße 2, Freising-Weihenstephan, 85350, Germany; 3 Research Institute, E. McMaster Building, The Hospital for Sick Children, Toronto, Canada; 4 Current address: Nestlé Institute of Health Sciences SA, EPFL campus, Quartier de l’innovation, Building G, Lausanne, 1015, Switzerland

**Keywords:** Inflammation, Inflammatory bowel disease, Eicosanoids, Eicosapentaenoic acid, Docosahexaenoic acid, Omega-3 fatty acids

## Abstract

**Background:**

Inflammatory bowel diseases (IBD) are chronic intestinal inflammatory diseases affecting about 1% of western populations. New eating behaviors might contribute to the global emergence of IBD. Although the immunoregulatory effects of omega-3 fatty acids have been well characterized in vitro, their role in IBD is controversial.

**Methods:**

The aim of this study was to assess the impact of increased fish oil intake on colonic gene expression, eicosanoid metabolism and development of colitis in a mouse model of IBD. Rag-2 deficient mice were fed fish oil (FO) enriched in omega-3 fatty acids i.e. EPA and DHA or control diet for 4 weeks before colitis induction by adoptive transfer of naïve T cells and maintained in the same diet for 4 additional weeks. Onset of colitis was monitored by colonoscopy and further confirmed by immunological examinations. Whole genome expression profiling was made and eicosanoids were measured by HPLC-MS/MS in colonic samples.

**Results:**

A significant reduction of colonic proinflammatory eicosanoids in FO fed mice compared to control was observed. However, neither alteration of colonic gene expression signature nor reduction in IBD scores was observed under FO diet.

**Conclusion:**

Thus, increased intake of dietary FO did not prevent experimental colitis.

## Background

Inflammatory bowel diseases (IBD) is a term used to cover a wide range of immune mediated diseases without a well defined etiology that result in chronic relapsing inflammation of the gut. The two major forms of IBD are Crohn’s disease (CD) and ulcerative colitis (UC). Genetic as well as environmental factors such as diet or composition and activity of intestinal microbiota have been implicated in IBD pathogenesis [1]. Experimental colitis induced by adoptive transfer (AT) of syngenic naïve T cells into lymphopenic mice is a well established animal model for IBD sharing a number of clinical, genetic and immunological features with human IBD [2].

Research on the effect of dietary lipids on the immune system has met great interest in the last decade. Amounts, types of fat and active lipid metabolites such as eicosanoids have an impact on immune cell function [3]. Long chain (LC) n-6 polyunsaturated fatty acids (PUFA) found in vegetable oils might promote proinflammatory responses potentially detrimental for the host. In contrast, LC n-3 PUFA found in fish oil (FO), specifically eicosapentaenoic acid (EPA) and docosahexaenoic acid (DHA), have been reported to support anti-inflammatory responses and thus have gained interest in the food industry. It is believed that the potential anti-inflammatory properties of LC n-3 PUFA may translate into important health benefits [4]. Indeed, epidemiological studies have revealed that the western populations, under high dietary ratios of n-6:n-3 PUFA, are more prone to develop chronic inflammatory diseases [5]. Nowadays, many dietary interventions target reduction of n-6:n-3 PUFA dietary ratio by introduction of marine products in the diet or n-3 PUFA supplements. However, pre-clinical studies provide inconsistent results on the anti-inflammatory properties of n-3 PUFA [6-20]. Indeed, in some studies n-3 PUFA display strong or mild effects on animal model of IBD [6-9,12,13,15-18,20] and in some other recent reports it was shown that a large dietary intake of n-3 PUFA could even exacerbate colitis [10,11,14,19]. The same discrepancies exist in clinics while recent systematic reviews and meta-analyses conclude that the available data are insufficient to draw any conclusions on the benefit of increased LC n-3 PUFA consumption for induction and/or maintenance of remission in IBD patients [21-24]. In contrast, clinical trials in ICU patients or in patients with rheumatoid arthritis have consistently demonstrated the anti-inflammatory efficacy of LC n-3 PUFA intake as recently reviewed in [25].

The use of “omics” technologies for systems biology with relevant animal models allow us to better understand the basic molecular mechanisms of how foods or food components like LC n-3 PUFA could prevent or ameliorate a disease such as IBD. Herein, using “omics” technologies and the well characterized mouse adoptive transfer (AT) colitis model [2,26], we have evaluated the properties of a LC n-3 PUFA-enriched diet under healthy or inflammatory conditions and determined whether LC n-3 PUFA-enriched diet may have a positive impact on colitis prevention. Colonic mucosa gene expression and eicosanoid metabolism were analyzed by transcriptomics and eicosanomics. Altogether our results based on a colitis mouse model allowed us to challenge the concept of efficacy of increased dietary intake of LC n-3 PUFA with FO for the prevention and management of IBD.

## Results

### Diet design and safety evaluation

**Figure 1:**
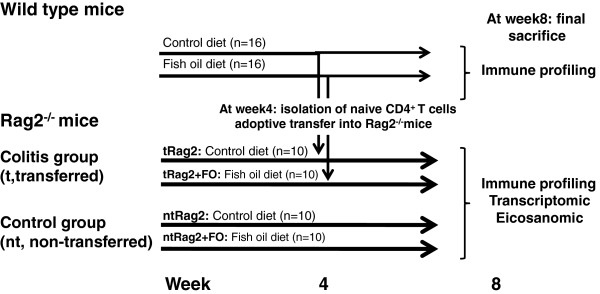
Study design.

In many studies, dietary oils are manipulated in order to modify ratios of n-6:n-3 PUFA. Often, high proportion of FO introduction leads to an increase in LC n-3 PUFA by increasing EPA and DHA intake while LC n-6 PUFA intake is reduced. Thus, the anti-inflammatory effects of FO may result from reduced cell content in LC n-6 PUFA like linoleic acid (LA) or arachidonic acid (AA) rather than the effect of EPA and DHA per se. Herein, in order to avoid this as a confounding factor, our diet described in Table 1 was designed to maintain LC n-6 PUFA and balanced for essential FA content in the control and experimental diets as analyzed and shown in Table 2. When wild type (WT) mice received this diet for 8 weeks no safety issue was noticed, all animals ate and grew normally. Immune cell subsets analysis (proportion and phenotype) by flow-cytometry in primary (thymus and bone marrow) and secondary lymphoid tissues (spleen) did not show any perturbation of myeloid cells, B and T lymphocyte development and function (data not shown). Therefore we decided next to test this diet in a colitis protocol established earlier for colitis prevention [2,26,27]. It was given to Rag2^−/−^ immune-deficient mice over a 4 week period before inducing IBD by AT of naïve T cells. AT mouse model of colitis was chosen because it is a well established animal model for IBD sharing a number of clinical, genetic and immunological features with the human disease [2,26]. The animals were kept under the same diet and followed for an extra 4 week period (see study design in Figure 1).

**Table 1:** Composition of the control and experimental diets (in gram per kilogram of dry matter)

**Nutrients (g/kg)**	**Control diet**	**(Experimental) FO-diet**
Corn Starch	549.5	549.5
κ-Casein	200	200
Sucrose	100	100
Cellulose	50	50
Mineral mix AIN-93 M	35	35
Vitamin mix AIN-93 M	10	10
L-cysteine	3	3
Choline bitartrate	2.5	2.5
Butylhydroxytoluene	0.014	0.014
Total oil mixture	50	50
Corn oil (%)	35	0
Cocoa butter (%)	15	7
Soybean oil (%)	50	50
Sunflower oil (%)	0	23
Fish oil (%)	0	20

**Table 2:** Fatty acid composition of the control and experimental diets

**Fatty acid**	**Control diet**	**(Experimental) FO-diet**
C14:0	0.10	1.49
C16:0	13.17	11.94
C16:1	0.32	1.99
C18:0	7.72	5.86
C18:1	25.67	21.06
C18:2n-6	47.72	42.66
C18:3n-3	4.34	4.17
C20:0	0.42	0.56
C20:1	0.01	0.13
C20:4n-6	0.00	0.22
C20:5n-3 EPA	0.00	3.37
C22:0	0.29	0.28
C22:5n-3	0.00	0.37
C22:6n-3 DHA	0.00	2.10
Other	0.24	3.81

Results of duplicate analysis are expressed as% of total fatty acids per gram of diet (dry matter).

### Colitis outcomes

**Figure 2:**
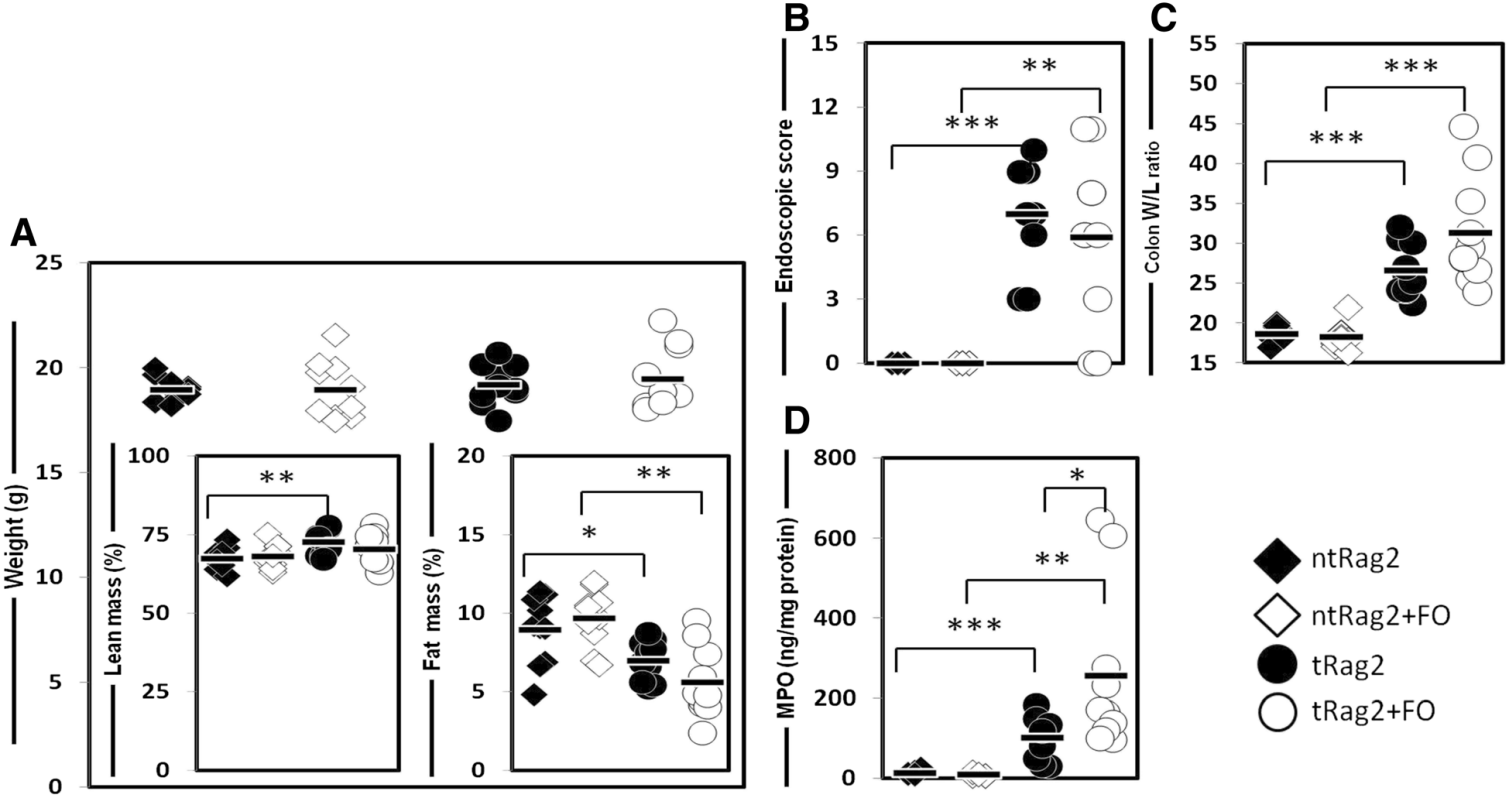
**Measures of colitic outcomes 4 weeks after AT.** Shown are body weight measures in grams at sacrifice and in inset shown is relative body composition with% lean mass in left panel and% fat mass in right panel (**A**). Endoscopic score is shown (**B**). Colon weight/length (W/L) ratio is shown (**C**). MPO content measured by ELISA is depicted (**D**). Black bars are median values and each dot represents the value obtained with an individual mouse. Statistics are shown as * when *P* <0.05, ** when *P* <0.01 and *** when *P* <0.001.

Following AT, colitis usually develops within 3–7 weeks and gets worse over time. To minimize animal suffering and strictly focus on IBD prevention by dietary intervention, we decided to stop at 4 weeks and focus on early signs of intestinal inflammation. At the time of sacrifice, as observed in Figure 2A, there was no significant weight loss when comparing the transferred (t) and the non-transferred (nt) animals. However, AT mice display a 22% reduction in fat mass (ntRag2 vs tRag2 *P* = 0.029, Figure 2A, inset) as previously observed as an early hallmark of IBD onset [28]. Fat mass loss was even more pronounced in FO fed AT animals reaching ~45% fat mass reduction (Figure 2A, inset ntRag2 + FO and tRag2, *P* = 0.001). Relative increased fat mass loss in tRag2 + FO compared to tRag2 was close to being significant (*P* = 0.089). The development of colitis upon AT was confirmed with macroscopic parameters such as endoscopic evaluation (Figure 2B, median scores of 7 and 6, tRag2 and tRag2 + FO, respectively) and an increased colon weight to length (W/L) ratio of 40-70% (Figure 2C), which was associated with a 4–13 fold increase in median spleen weight and cellularity (data not shown). However none of the macroscopic parameters of colitis showed significant differences between control-fed and FO-fed animals (Figure 2B-C).

**Figure 3:**
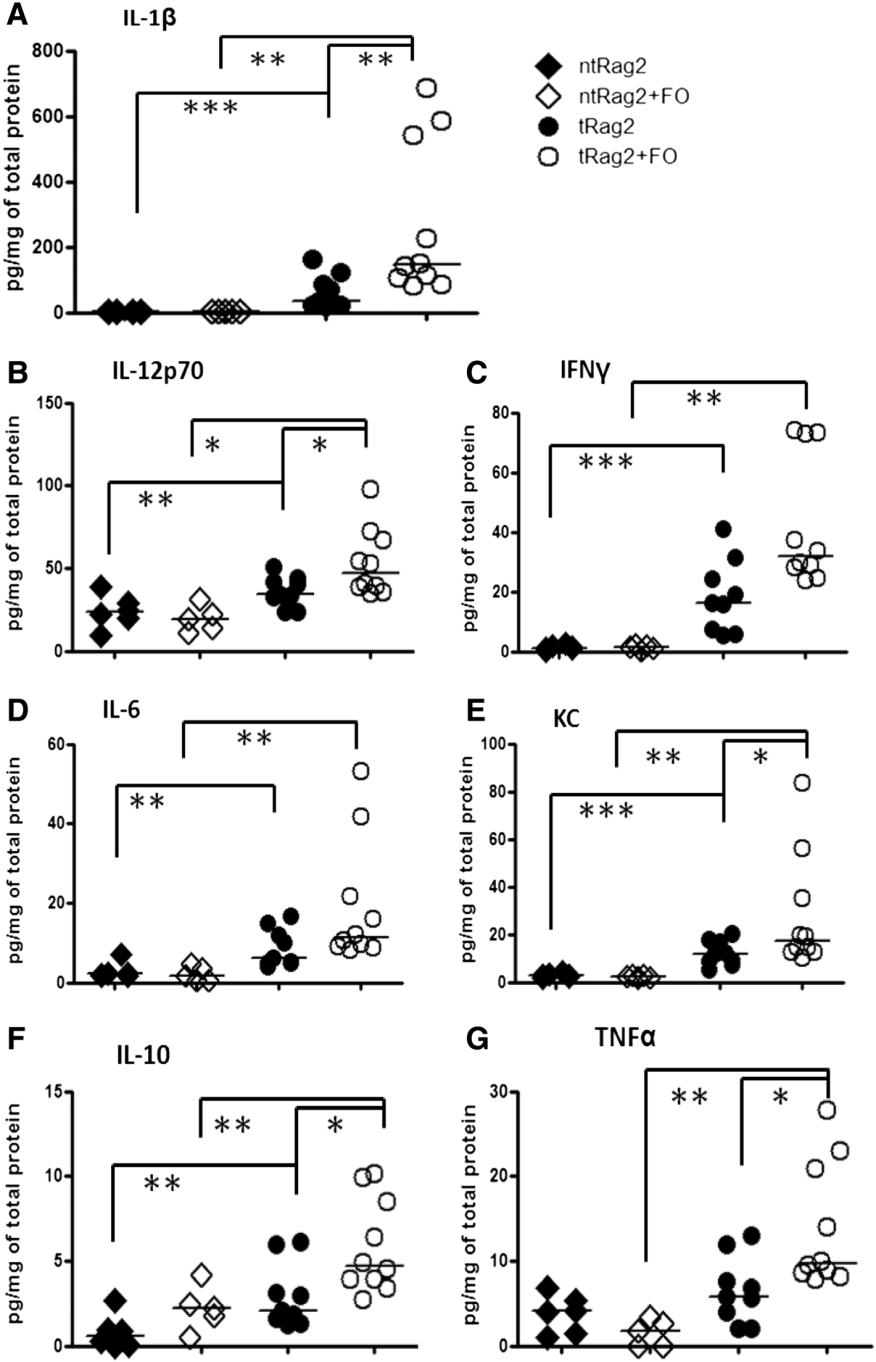
**Cytokine measurements in colon tissue.** IL-1β; IL-12p70; IFNγ; IL-6; KC; IL-10 and TNFα are shown (from **A**-**G** respectively). Black bars are median values and each dot represents the value obtained with an individual mouse. Statistics are shown as * when *P* <0.05, ** when *P* <0.01 and *** when *P* <0.001.

**Figure 4:**
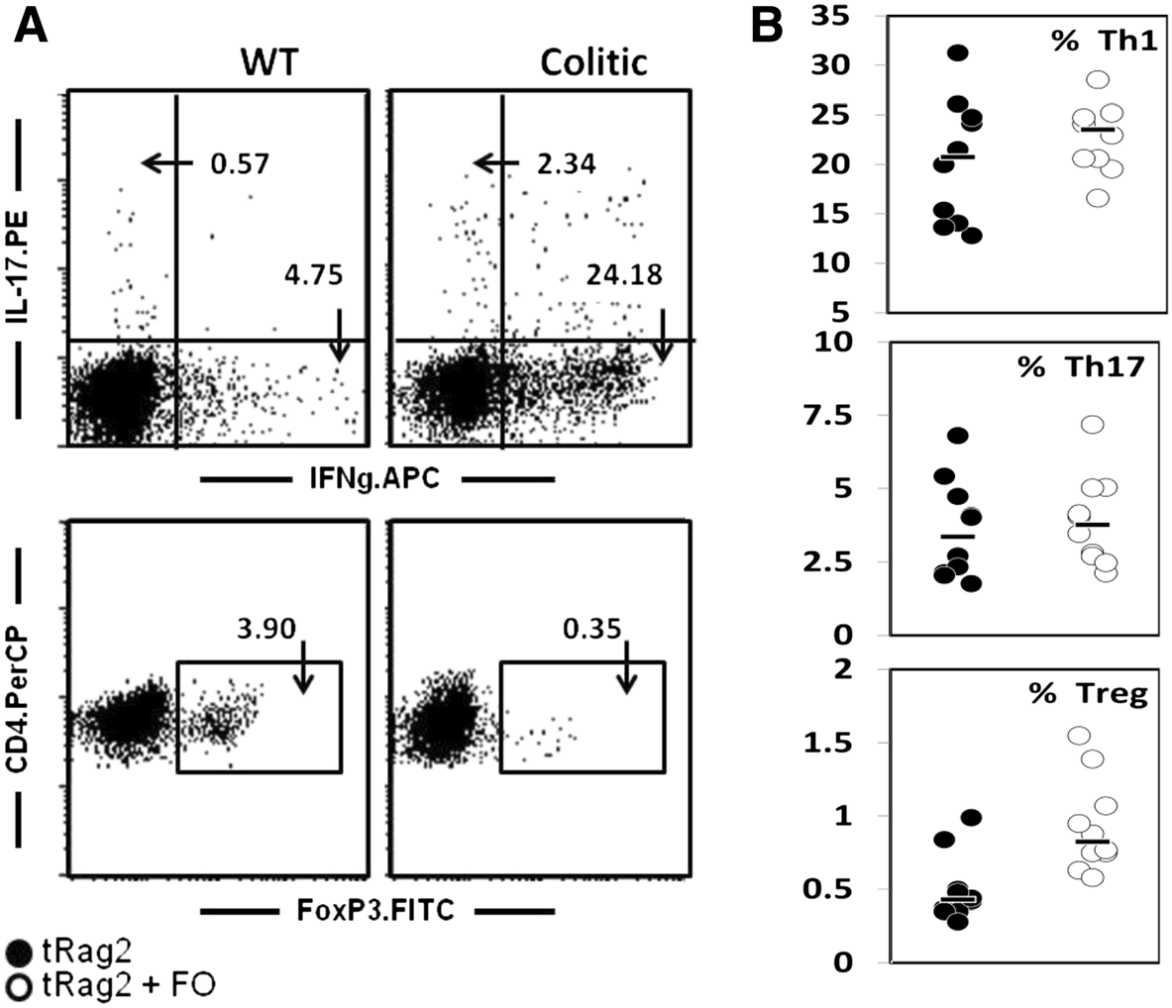
**Phenotypic analysis of T-helper cells in mesenteric lymph nodes at 4 weeks after AT.** Ex-vivo T cell stimulation, fixation and staining were performed then analyzed by flow-cytometry. Representative flow-cytometry dot plot pictures and gating strategy to quantify% of Th1, Th17 and regulatory T cells (**A**). Note the clear differences in each subset frequency between a healthy mice and colitic mice irrespectively of the diet used.% of Th1, Th17 cells and regulatory T cells are shown in colitic mice (tRag2 and tRag2 + FO) (**B**). Black bars are median values and each dot represents the value obtained with an individual mouse.

In addition to the macroscopic parameters, immune colitis hallmarks i.e. mucosal Th1/Th17 inflammatory disease with immune cell infiltrates were confirmed. Indeed a 10 fold increase in colonic MPO was shown in tRag2 vs. ntRag2 reflecting massive neutrophil infiltration (Figure 2D, *P* < 0.001), as well as significant increases of pro-inflammatory cytokines, mainly IL-1β, IL-6, KC and IFNγ (Figure 3, ntRag2 vs. tRag2). Surprisingly, FO-fed animals showed higher levels of colonic MPO (Figure 2D, tRag2 vs. tRag2 + FO, *P* = 0.028), IL-1β (Figure 3A, tRag2 vs. tRag2 + FO, *P* = 0.004), IL-12 (Figure 3B, tRag2 vs. tRag2 + FO, *P* = 0.035), KC (Figure 3E, tRag2 vs. tRag2 + FO, *P* = 0.035), IL-10 (Figure 3F, tRag2 vs. tRag2 + FO, *P* = 0.01) and TNFα (Figure 3G, tRag2 vs. tRag2 + FO, *P* = 0.006). Phenotypic and functional analysis of mesenteric lymph node T cells isolated from colitic mice confirmed the presence of IFNγ-secreting Th1 cells with about 24% of CD4^+^ T cells, and to a lesser extent IL-17 secreting Th17 pathogenic T cells with about 2-3% of CD4^+^ T cells, whereas the FoxP3^+^ regulatory T cells were almost absent <1% of CD4^+^ T cells (Figure 4). In addition, supernatants of anti-CD3 + anti-CD28 or LPS ex vivo stimulation contained higher level of the same above proinflammatory cytokines (data not shown). Altogether our data shows that increased intake of FO neither altered mucosal pathogenic T cells development nor prevented colitis development.

### Colonic eicosanomics

**Figure 5:**
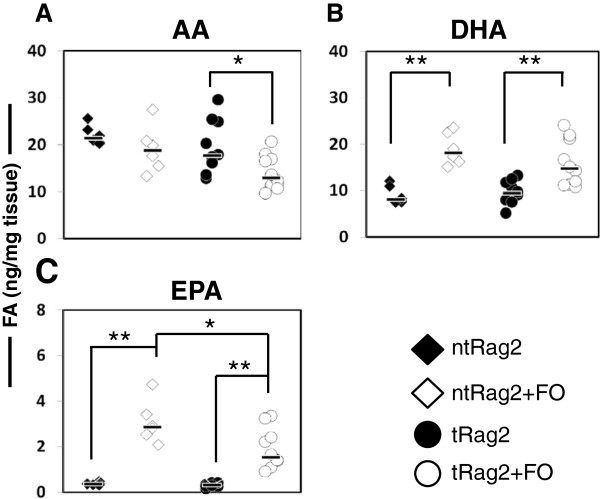
**Colonic free FA levels of AA, DHA and EPA.** AA, DHA and EPA levels are shown for each experimental group (from **A**-**C** respectively). Black bars are median value and each dot represents the value obtained with an individual mouse. Statistics are shown as * when *P* <0.05, ** when *P* <0.01 and *** when *P* <0.001.

EPA and DHA are described as the most important anti-inflammatory FA contained in FO. Therefore, as no or few unexpected differences were observed under FO consumption, we next wanted to confirm that our dietary intervention does indeed increase the content of EPA and DHA in healthy animals (ntRag2 vs ntRag2 + FO) and check AA content as well. After 8-week feeding animals were sacrificed and AA, DHA and EPA levels were measured as total free FA in the target tissue i.e. colon. As shown in Figure 5A, the median AA levels were at 21.4 ng/mg tissue in the ntRag2 mice while they were 18.7 ng/mg tissues in the ntRag2 + FO mice. This difference was not significant. On the other hand, median AA levels in the T cell transferred mice, i.e. tRag2 and tRag2 + FO mice were 17.7 and 13 ng/mg tissue respectively (*P* = 0.03). In contrast and as expected, median levels of DHA shown in Figure 5B were at 8.1 and 18.1 ng/mg tissue in the non transferred mice, i.e. ntRag2 and ntRag2 + FO (*P* = 0.002) and 9.4 and 14.7 ng/mg tissue in the transferred mice, i.e. tRag2 and tRag2 + FO (*P* = 0.002). Similarly, EPA levels shown in Figure 5C were also increased after dietary intervention in both the non-transferred and the transferred animals median levels were 0.4 and 2.9 ng/mg tissue in the non transferred mice, i.e. ntRag2 and ntRag2 + FO (*P* = 0.002) and 0.3 and 1.6 ng/mg tissue in the transferred mice, i.e. tRag2 and tRag2 + FO (*P* < 0.001). Overall, we observed an increase in the median levels of free colonic EPA and DHA by up to 7.2 and 2.2 fold, respectively. Despite reaching a similar level of AA with both diets in the non-transferred, a small reduction in AA (0.73 fold) was observed only in transferred mice which might result from an increased use induced by the inflammatory conditions.

**Figure 6:**
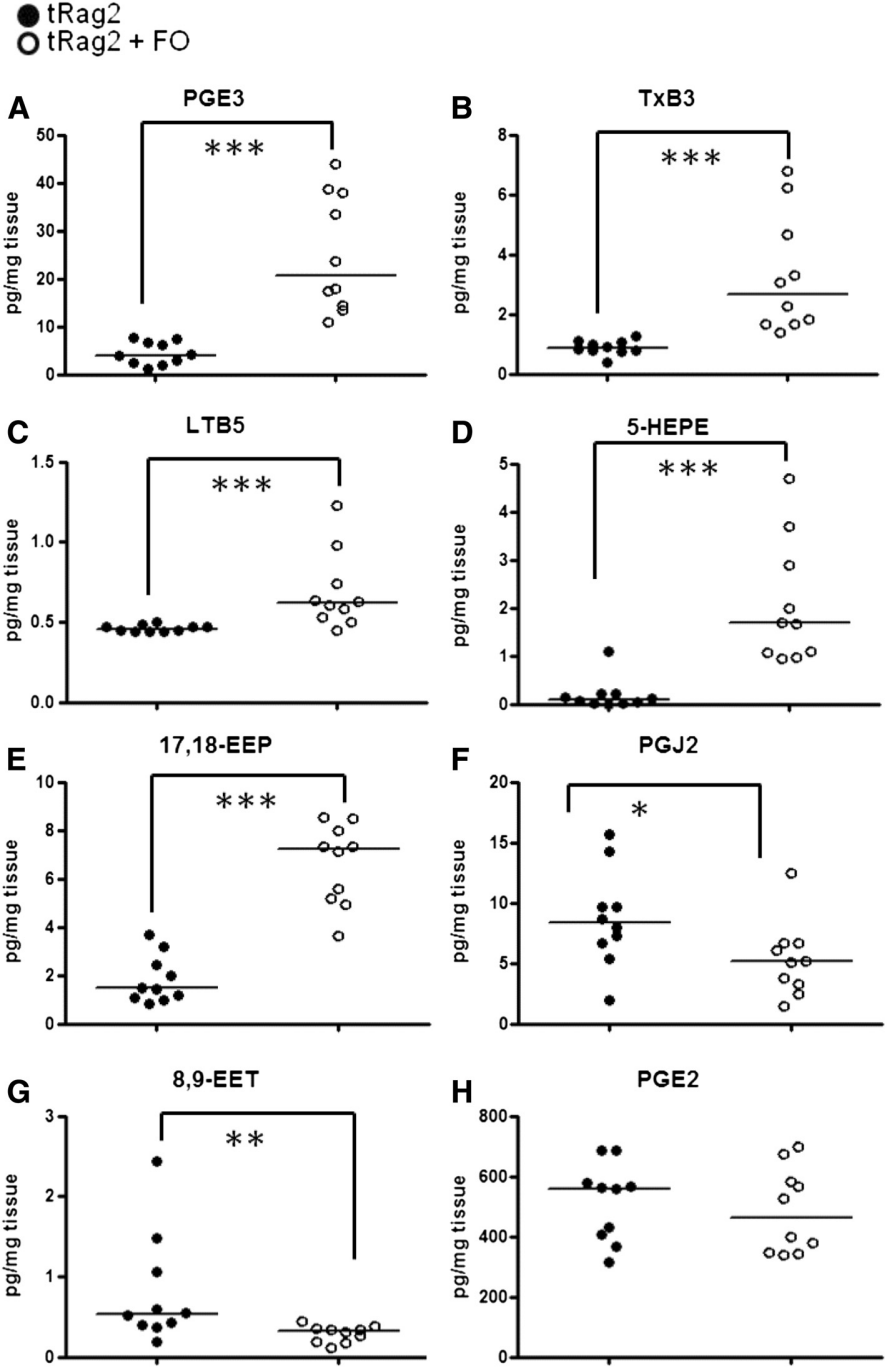
**Levels of EPA and AA-derived metabolites in colon.** Free fatty acid levels of EPA-derived metabolites prostaglandin E3 (PGE3); thromboxane B3 (TxB3); leukotriene B5 (LTB5); 5-hydroxyeicosapentaenoic acid (5-HEPE); 17,18-epoxyeicosatetraenoic acid (17,18-EEP); and the AA-derived metabolites prostaglandin J2 (PGJ2); 8,9-epoxyeicosatrienoic acid (8,9-EET); prostaglandin E2 (PGE2) (from **A**-**H** respectively) are shown. Black bars are median values and each dot represents the value obtained with an individual mouse where dark circles are tRag2 and open circles are tRag2 + FO. Statistics are shown as * when *P* <0.05, ** when *P* <0.01 and *** when *P* <0.001. Of note whole set of metabolites measured and corresponding values are given in Additional files 1 and 2.

The beneficial effects of EPA and DHA are generally ascribed to the increase in EPA- and DHA-derived metabolites instead of AA-derived ones. Thus, we developed a method to investigated the generation of 22 AA- and 19 EPA/DHA-derived metabolites i.e. eicosanoids in the colon. The levels of 8 relevant selected AA- and EPA/DHA- derived metabolites under colitic conditions with the control and experimental diets are depicted in Figure 6, while a whole list of 41 metabolites measured and respective values and statistics are given in Additional files 1 and 2. In accordance with the changes in the precursors, most of the EPA- and DHA- derived metabolites described as anti-inflammatory molecules increased following FO diet. We confirmed the release of the precursors as well as the formation of the COX (prostaglandin (PG)E3 and thromboxane (Tx)B3, Figure 6A-B), LOX (leukotriene (LT) B5 and 5-hydroxyeicosapentaenoic acid (5-HEPE), Figure 6C-D) and CYP450 (17,18-epoxyeicosatetraenoic acid (17,18-EEP), Figure 6E) derived metabolites of EPA and DHA in the colon following our dietary intervention. With respect to AA-derived metabolites, despite a significant decrease in free AA in the colon, only a few metabolites were reduced, namely PGJ2 (Figure 6F), 5,6-epoxyeicosatrienoic acid (5,6-EET), 8,9-EET (Figure 6G) and 14,15-EET. Thus, the decrease in free AA did not translate into a reduction of important proinflammatory mediators such as PGE2 (Figure 6H), TXB2 and LTB4 (Additional files 1 and 2). Furthermore, it should be noted that the absolute amounts of the series 2 and the series 3 metabolites, arising from AA and EPA, respectively, differed by almost 1 order of magnitude in favour of series-2 metabolites indicating that there was not a substantial redirection of eicosanoid expression into series-3 metabolites in this experimental model.

### Colonic whole gene expression profiling

**Figure 7:**
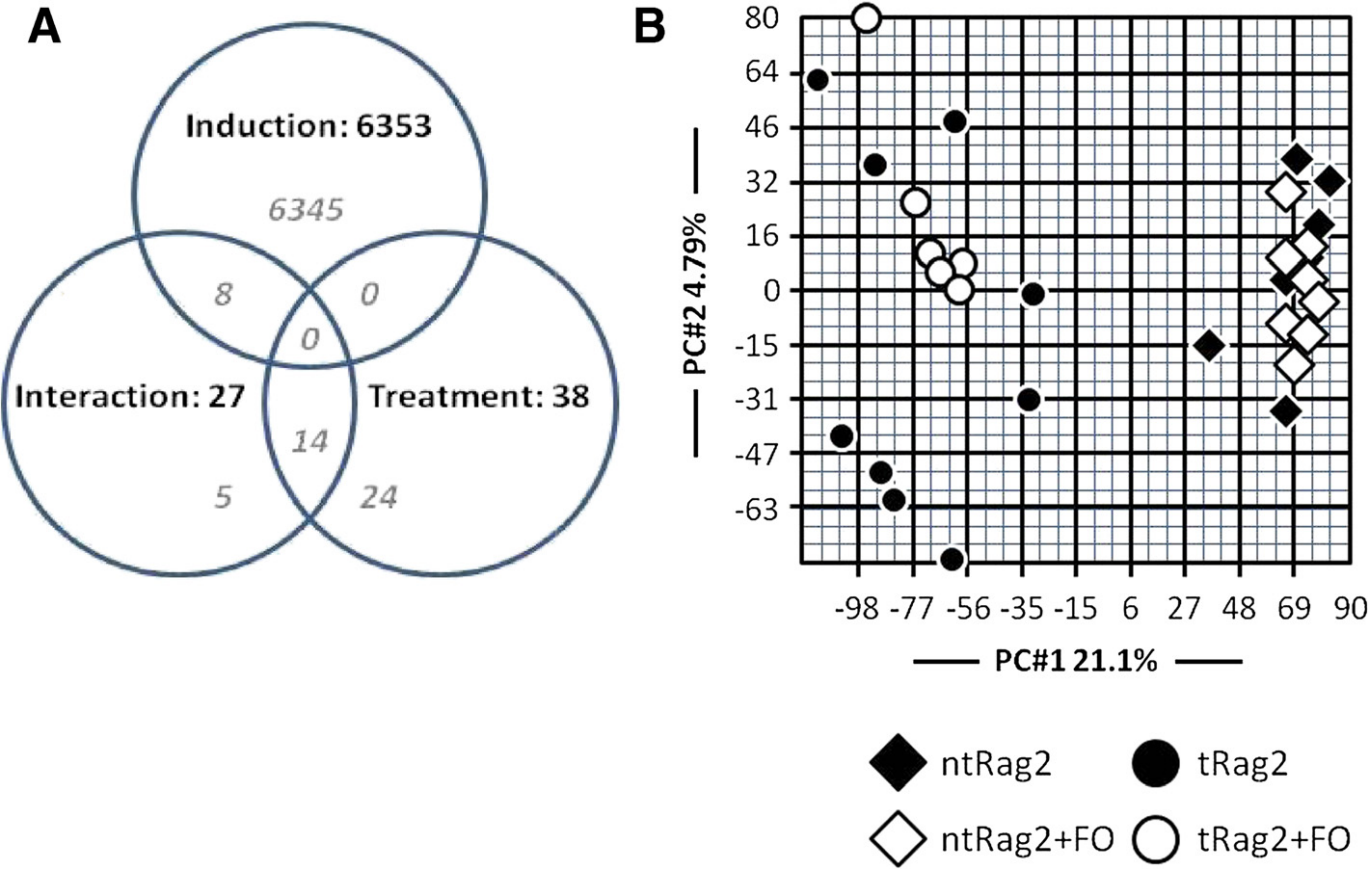
**Transcriptomic analysis of colon preparation in healthy and colitic mice.** Shown is Venn diagram made with significantly regulated colonic genes (*P* < 0.001) showing the relative impact of diet (treatment) vs. colitis (induction) and interaction between diet and colitis (**A**). Two-dimensional visualization of unsupervised PCA analysis constructed with the whole set of gene significantly differentially expressed in the colon upon colitis induction (**B**). Each dot represents the value obtained with an individual mouse (n = 6-9 mice per group). Of note top 40 colonic genes up- and down regulated in colitis mice and corresponding value are given in Additional files 3 and 4.

An earlier transcriptomic study with FO intervention had shown inhibitory effects on nuclear factor-κB (NF-κB) regulated genes in human PBMCs [29]. Thus we wanted to determine whether colitic processes are modulated by FO diet in the target organ i.e. colon. RNA from colonic tissue was isolated 28 days after AT and subjected to genome-wide screening. Microarray analysis identified 6353 probes that were differentially expressed between healthy and colitic mice (Figure 7A). Among them, 215 and 50 genes were up- or down-regulated respectively by >2 fold. Mucosal inflammatory processes are evidenced by top 40 up- and down-regulated genes found in colon mRNA preparation of colitis mice (Additional files 3 and 4). We observed important increased expression of a set of genes belonging to proinflammatory cytokine (IL-1β, 4 fold), chemokine family (CXCL9, CCL5, CCL8, CXCL10 and CCL4 up to 22.4 fold) or acute phase proteins (SAA1, 7 fold). It explained massive leukocyte infiltration supported by high expression of neutrophil related genes (formyl peptide receptor (FPR)-2 or CD16a up to 10.3 fold) or T cell specific gene (CD3γ and CD52 up to 4.8 fold). Epithelial cell activation is also evidenced by raised expression of major histocompatibility complex (MHC) class I or II or costimulatory molecules (i.e. H2 molecules, CD74 known as MHC-II invariant chain and CD274 known as PD-L1 or B7-H1) up to 6.4 fold as well as intestinal anti-microbial peptide expression (S100A8, lysozyme 2 and Reg3α up to 6.4 fold). Interestingly these expression patterns have also been reported in human or animal studies using different models of IBD namely DSS or TNBS mediated intestinal inflammation [2,30]. In parallel, as a reflection of the mucosal functional impairment associated with colitis, we also observed about a 2–3 fold reduced expression of a set of genes reported to play major a role on water (*aqp1*-aquaporin 1 [31]), electrolytes (*slc24a3-*solute carrier family 24 member A3 being a Na^+^/K^+^/Ca_2_^+^ exchanger [32] or *FXYD4* or *FXYD6*-FXYD domain containing ion transport regulator 4 or 6 [33]) or vitamin (*slc5a6 –* solute carrier family 5 member A6 being a Na^+^-dependent multivitamin transporter [34]) absorption. In addition, in colitic mice, despite increased FO intake, some important genes involved in endothelial smooth muscle function (e.g. *actg2*–gamma 2 enteric smooth muscle actin [35], *synm*-synemin also known as desmuslin [36], or *myh11*–smooth muscle myosin heavy chain 11 [37]) were down-regulated by up to 2.3 fold. Interestingly *mptx* gene -mucosal pentraxin- a previoulsly reported rodent-specific biomarker of gut health [38] was also decreased by >2 fold. Overall, this might reflect general alteration of colonic tissue histology and function.

Importantly, only 38 probes were differentially regulated by dietary intervention as shown in Venn diagram (Figure 7A). As mentioned in the methods, the analyses were performed at an alpha of 0.001 which yielded a false positive rate of ~26. Further, while more than 2000 probes were changed by more than 25% with colitis, no probes were altered by more than 25% specifically by the diet. Thus, we believe that the impact of FO was negligible under these conditions in the inflamed colonic tissue. In agreement with this observation, global unsupervised analysis did not reveal any effect of FO diet on inflammation as visualized by PCA analysis on the total set of 25697 probes analyzed (Figure 7B).

## Conclusions

Our dietary intervention study was aimed at increasing cellular EPA and DHA content while maintaining normal AA levels and to test whether this prevents features of colitis development in a mouse model of IBD. Despite increased dietary intake of n-3 PUFAs via FO and efficient colonic incorporation of EPA and DHA, we did not observe protection against colitis onset in our mouse model 4 weeks after disease induction. The differences between our studies and others might be explained by multiple variables like diet, species, and the nature and or severity of the IBD model used. Herein, we carefully established a diet maintaining total n-6 PUFA content in both control and experimental diet, in order to strictly examine the impact of increased intakes of n-6 PUFA i.e. EPA and DHA rather than the relative decrease of AA observed in many studies which can be a confounding factor. However, our diet did not convey anti-inflammatory activity or at least sufficient enough to prevent colitis. Indeed despite FO-based dietary intervention, in colitic mice mucosal proinflammatory chemokines and cytokines mRNA and protein levels were maintained or even exacerbated, pathogenic Th1/Th17 cells present and neutrophil infiltrates increased. As we fed animal before colitis induction and stopped our experiments 4 weeks, for internal ethical reasons, we cannot firmly anticipate subsequent positive impact or therapeutic properties of FO-based diet.

A recent study by Fenton and colleagues, using a different approach with *smad-*3^−/−^ mice infected with *Helicobacter hepaticus* as a colitis model [19], showed that inflammation severity and dysplasia was positively correlated with the amount of FO present in the diet, the reduction of CD8^+^ T cell frequency and the increase in regulatory T cell frequency at week 4 post-infection. AT colitis model consists in syngeneic transfer of naïve CD4^+^ T cells into lymphopenic mice, thus CD8^+^ T cells and regulatory T cells are normally absent or very limited in numbers (due to poor contamination of inoculums). Transferred T cells dramatically expand and particularly in colon in response to microbiota non-self antigens due to empty space and lack of regulatory mechanisms provided by regulatory T cells, colitis develops without any need of infection [26,39]. These major differences might explain why in our settings FO-diet did not dramatically exacerbate the colitis. The impact of an infection subsequently to colitis induction by AT might be interesting to study as well as the effects of fish oil in such settings.

Herein, we studied for the first time the combined effect of dietary n3-PUFA and or inflammation on colonic eicosanoid metabolism in vivo. Efficient incorporation of EPA and DHA in the colonic tissue as well as generation of EPA- and DHA-derived anti-inflammatory metabolites were confirmed through eicosanomic analysis. A “favorable” lipid signature was found but unfortunately not enough to identify a cause to prevent colitis as one would have expected. Very similar conclusions were obtained in mouse models of arthritis by Lyme infection or sepsis [40,41]. Brown and colleagues managed to significantly shift eicosanoid profile in arthritic ankle joints by substitution of soybean oil with FO in the diet but did not change host inflammatory response or development of arthritis [40]. The same is true for sepsis as Witkamp and collegues showed that FO intake generally increased series-3 eicosanoids but failed to improve septic signs and detect resolvins [41]. Of note, mice who received higher dose of FO displayed relatively more severe signs of sepsis.

We observed that absolute amounts of the series-2 eicosanoids arising from AA and the series-3 eicosanoids arising from EPA, differed by almost 1 order of magnitude (Additional files 1 and 2). Therefore, we assume that despite high dietary intake, it might be difficult or even impossible to counterbalance proinflammatory effects of series-2 eicosanoids competing for the same receptors as anti-inflammatory series-3 eicosanoids in physiological situation. Therefore in some settings where this balance can be efficiently impaired like *fat*-1 transgenic animals, better inflammatory outcomes are obtained [42-44]. In contrast to unmanipulated mammals where n-3 PUFA almost exclusively come from diet (as herein), *fat-1* transgenic mice expressing *C. elegans fat-1* gene i.e. an n-3 fatty acid desaturase and can convert dietary n-6 PUFA into n-3 PUFA in all tissues. Those mice were protected against TNBS- or DSS-induced colitis. Two protective mechanisms were described. First it leads to a significant increase in anti-inflammatory lipid metabolites derived from n3-PUFA namely protectin D1, resolvin E1 and D3 whereas pro-inflammatory lipid metabolites derived from n6-PUFA like LTB4 and PGE2 were kept constant [42,43]. More recently Chapkins and colleagues showed that colonic mucosal microenvironment was altering regulatory T cells/Th17 cells ratio in *fat-1* mice [44]. These data highlight the importance of the nature of the anti-inflammatory lipid metabolites but also their relative amounts in target tissue. Eicosanoids not only play a role on inflammation, they could also support mucosal pathogenic Th17 cells generation [45]. In our experimental condition, we neither observe alteration in helper T cell numbers and subsets nor detect resolvins in line with the lack of efficacy of our dietary FO treatment. Overall n3-derived lipid metabolites were 10–100 fold lower than their n-6 counterparts. This important bias in n-3:n-6 eicosanoid stoichiometry might explain why colitis amelioration was not achieved despite the substantial EPA and DHA precursor dose provided within the diet. In that respect, new tricks that could be translated to human clinics, to improve n3-derived lipid metabolites generation or n3:n6 ratio in vivo deserve further investigations. This could be achieved by the concomitant use of dietary FO with phospholipase A2 inhibitors (to reduce AA release and generation of proinflammatory eicosanoids [46]) and or COX-2 inhibitors (to promote resolvins generation [47]).

To fully complete our work and gain larger insight into the effect of dietary EPA and DHA on colonic inflammation, microarray analysis was performed with tissue from healthy or colitic mice under control or experimental diet. Multivariate data modeling validated our previous findings showing limited effects on colonic gene expression induced by FO consumption. Indeed, expression levels of proinflammatory markers, e.g. chemokines and acute phase proteins, and epithelial cell stressors were unaffected. As this observation was made out of global tissue rather than cell specific examination, we cannot rule out that dietary EPA and DHA control gene expression profiles in a tissue and cell specific way. For instance, significant impact of similar amount of dietary EPA and DHA on liver or PBMC gene expression profile was previously documented [29,48]. Herein, we demonstrate that colon gene expression profile is less influenced by dietary LC n-3 PUFA. Further examinations of colon epithelial cells or intra epithelial lymphocytes deserve further investigations.

We reported new important intestinal events associated with colitis which might explain morphological and functional alteration of the colon. For instance, we observed a significant reduction in *slc5a6* gene expression coding for Na^+^-dependent multivitamin transporter. SLC5A6 is a crucial transporter of biotin, a water-soluble vitamin required for normal cellular function, growth and development [34]. Intestinal-specific deletion of *slc5a6* was recently reported in mice [34]. Two-thirds of the intestinal *slc5a6*^−/−^ mice died prematurely due to acute peritonitis. The remaining mice displayed important reduction of blood biotin level associated to growth retardation and specific intestinal inflammation and histological alterations. In that respect, vitamin status in preclinical model of IBD or patients, which often display multiple vitamin deficiencies [49] deserves better attention as well as specific means to restore normal levels.

How can this data be further translated to human? IBD is a disease with remission-relapse periods where induction and maintenance therapies should be considered. Our model like others does not address properly this dynamic feature of IBD as a relapsing disease with inflammatory and healing episodes. However, clinical trial results assessing the role of FO in both stages exist and also ruled out potential benefits of EPA and DHA in induction or maintenance therapies [22-24,50]. Concerning the dose provided to the animal, we estimate that about 10 mg EPA + DHA per animal per day were consumed. It represents a human equivalent dose (HED) [51] of about 41 mg per kg of body weight per day i.e. about at least 3 supplement pills (of the highest marketed concentration) or ~250 grams daily consumption of wild salmon in healthy adults of average body weight of 70 kg. Such a high dose is close to the highest daily intake recorded in epidemiological studies where EPA + DHA dietary intake was associated with IBD disease reduction risk [52]. However, no efficacy was shown herein as well as in clinical trials. Thus, no firm recommendations about the usefulness of n-3 LC-PUFAs can be made for UC or CD patients. We also believe that important genetic or environmental factors not addressed in epidemiological studies might at least partially contribute to the preventive action attributed to n3-PUFA against IBD.

In summary, our results show that increase intake of dietary n-3 PUFA in mice does not reduce colitis development in AT colitis model. Transcriptomic analysis reveals a limited impact of these dietary lipids on IBD. In contrast, eicosanomic analysis reveal significant increase of some anti-inflammatory colonic eicosanoids when mice where fed with FO. However, even though some of these mediators might play a positive role in colitis prevention, their presence in limited amount relative to the proinflammatory mediators derived from AA was not sufficient to alleviate colitis.

## Materials and methods

### Animals, housing and diets

Wild-type (WT) or Rag2^−/−^ C57BL/6 breeder mice were purchased from CDTA Orleans (France). Breeding was maintained in specific pathogen-free conditions at Nestlé research center animal care facility then transferred to conventional housing conditions and kept in ventilated cages for our experiments. Animals had free access to diet and tap water. Control and experimental diets composition are given in Table 1 and based on a standard AIN-93G rodent diet then FA composition of both diets was checked by classical methods as described previously [53] and given in Table 2. Based on an average mouse body weight of 20 g and 4 g of daily food intake, our mice consumed about 10 mg per day of EPA + DHA. This dose is equivalent to ~41 mg/kg/day in humans according to the human equivalent dose formula (HED) calculated as HED_(EPA+DHA)_ = animal dose in mg/kg × (animal weight in kg/human weight in kg)^0.33^[51]. Powders were transformed into pellets, dried at low temperature and stored in small sachets under vacuum at −20°C. The diets were changed twice a week in each animal cage. These precautions were taken to avoid oxidative degradation of lipids. Female mice between 8–12 week old were used. WT or Rag2^−/−^ animals were fed either the control or experimental diet 4 weeks prior to colitis induction and under the same diet for 4 additional weeks as depicted in study design (Figure 1). All experiments were conducted according to the Nestlé animal welfare policy and approved by Swiss governmental veterinary offices (authorisation number VD-2076.1).

### Colitis induction

At the time of colitis induction WT mice (n = 10) from each group (control or experimental diet) were euthanized, 5x10^5^ naïve CD4^+^CD25^-^CD45RB^High^ T-cells were isolated and i.p. transferred into Rag2^−/−^ mice to induce colitis as described previously [2,26,27,39]. The remaining WT mice and the Rag2^−/−^ mice, both transferred (t) and non-transferred (nt), were further fed the control and experimental diets as depicted in Figure 1 for another 4 weeks a timing previously established for first signs of IBD apparition [27]. Along the 4 week period post-transfer, mice were observed for clinical signs of well-being and illness. At sacrifice (day 28–29 post transfer), colons were removed from ileo-cecal junction to rectum, cleaned with cold PBS then weight and length were measured before being snap-frozen in liquid nitrogen.

### Colonoscopy and body composition

The COLOVIEW mini-endoscopic system was used as previously described [54]. Distal colon was examined along the first 3–4 cm. Scoring system [0–30] consists in evaluation of ulceration numbers (0–6), vasculature features (0–3), mucosal granularity (0–3), erythema (0–3), pinpoints (0–3), fibrin deposition (0–3), length involved (0–6) and overall vulnerability (0–3) of the colon. Fat and lean body mass were measured with NMR (EchoMRI 2004) the day before the sacrifice and expressed as% of animal body weight.

### Helper T cell (Th) and regulatory T cell characterization

Mesenteric lymph node cell suspensions were made to assess in AT mice Th1 and Th17 cells ex vivo as described previously [27]. Th1 cells were CD4^+^IFNγ^+^ whereas Th17 cells were CD4^+^IL-17^+^. Additionally, anti-FoxP3 intranuclear staining was made in order to track the generation of so-called CD4^+^FoxP3^+^ regulatory T cells. All antibodies were purchased from eBiosciences.

### Cytokines and myeloperoxidase (MPO) measurements

Ultrasensitive multiplex cytokine profiling kit (Meso Scale Discovery) was used to assess mouse IL-1β, IL-6, keratinocyte-derived chemokine (KC) (mouse IL-8), IL-10, IL-12p70, IFNγ and TNF-α in colonic protein extracts according to manufacturer’s instructions. Proteins from colon samples were prepared in RIPA buffer (Sigma) and protein measured with RC-DC Protein assay kit (BIORAD). MPO content of the colon protein extracts was determined with an ELISA kit (Hycult Biotech) following the manufacturer’s instructions. Cytokines or MPO levels were normalized to total tissue protein contents.

### Microarray analysis

As previously described [55], colon samples were homogenized in lysis buffer using a FastPrep instrument, in lysing tubes containing ceramic beads (MP Biomedicals, Irvine, CA, USA). Total RNA was extracted and purified with the RNAdvance tissue kit (Agencourt, Beverly, MA, USA). The quality of RNA samples was checked by using the Agilent 2100 Bioanalyzer (RNA integrity numbers ≥ 8 for high quality; Agilent Technologies, Santa Clara, CA, USA). All cRNA targets were synthesized, labeled, and purified according to the Illumina TotalPrep RNA amplification protocol (Applied Biosystems, Austin, TX, USA). Then, 15 μl of each hybridization mix was dispensed on the microarrays (16 h, 58°C), the microarrays were washed to remove non hybridized material and stained with Streptavidin-Cy3. All samples were analyzed with the microarrays MouseRef-8 v2 Expression BeadChips (Illumina, San Diego, CA, USA).

### Eicosanomics analysis

AA, EPA, DHA and their respective derived metabolites were quantified by HPLC-MS/MS as described earlier [53]. Calibration curves were generated from amounts of 10 pg to 1 ng of undeuterated standards (Cayman Chemical, USA) and a fixed quantity of deuterated internal standards (1 ng) for each analyte. Quantitation of analytes was done with the Analyst (1.5.1) software.

### Statistical analysis

Except microarray data, all the data presented herein were analyzed with the 2-sided Wilcoxon rank sum test with the R software, version 2.12.0. Differences were considered statistically significant for *P* value <0.05. Since this is non-parametric, median values were used. Differential gene expression obtained by between the groups with colitis induction and diet as factors was analyzed with a 2-way ANOVA followed by a-posteriori contrasts. Microarray gene expression data are presented as fold change for top-40 genes, based on the *P* value cutoff <0.001 and analyzed with the Ingenuity Pathways Analysis software (IPA; Ingenuity Systems).

## Abbreviations

AA: Arachidonic acid; AT: Adoptive transfer; CD: Crohn’s disease; EEP: Epoxyeicosatetraenoic acid; EET: Epoxyeicosatrienoic acid; FO: Fish oil; HEPE: Hydroxyeicosapentaenoic acid; IBD: Inflammatory bowel disease; LT: Leukotriene; PBMC: Peripheral blood mononuclear cell; rag2: Recombination activating gene 2; TX: Thromboxane; UC: Ulcerative colitis.

## Competing interests

NB, VB, MO, F-PM, FR, RM, SM, VBS, SR and JB are employees of Nestec SA.

## Authors’ contributions

NB, VB and MO designed the research. NB, VB, MO, F-PM, PL, FR, RM, SM and CP-A performed experiments and analyzed data. RM and VBS did the statistics. NB and VB wrote the paper. SR, DH and JB support and review the study. All authors read and approved the final manuscript.

## Supplementary Material

Additional file 1 **List of AA-derived metabolites quantified in colon and values.** Colon preparations of control and colitis animals under control- or FO-diet were analyzed. Medians are expressed in pg/mg of tissue. Significant differences between group comparisons are highlighted in grey. Click here for file

Additional file 2 **List of DHA- and EPA-derived metabolites quantified in colon and values.** Colon preparations of control and colitis animals under control- or FO-diet were analyzed. Medians are expressed in pg/mg of tissue. Significant differences between group comparisons are highlighted in grey. Click here for file

Additional file 3 **Top 40 up-regulated colonic genes.** Differentially expressed genes in colitis mice compared to control mice fed with control diet are shown. All with *P* < 0.001, with n = 8 and 9 mice for ntRag2 and tRag2 respectively. Illumina ID probe number is given. Click here for file

Additional file 4 **Top 40 down-regulated colonic genes.** Differentially expressed genes in colitis mice compared to control mice fed with control diet are shown. All with *P* < 0.001, with n = 8 and 9 mice for ntRag2 and tRag2 respectively. Illumina ID probe number is given. Click here for file
