# Supplementary material for: Effects of increase in fish oil intake on intestinal eicosanoids and inflammation in a mouse model of colitis
Source: Lipids Health Dis. 2013 May 31;12:81. doi: 10.1186/1476-511X-12-81 (PMC3691874; doi:10.1186/1476-511X-12-81)
Supplement: Additional file 1 — List of AA-derived metabolites quantified in colon and values. Colon preparations of control and colitis animals under control- or FO-diet were analyzed. Medians are expressed in pg/mg of tissue. Significant differences between group comparisons are highlighted in grey. [file 1476-511X-12-81-S1.docx]

**Additional file 1:** List of AA-derived metabolites quantified in colon and values.

Colon preparations of control and colitis animals under control- or FO-diet were analyzed. Medians are expressed in pg/mg of tissue. Significant differences between group comparisons are highlighted in grey.

| **Metabolite (pg/mg)** | **Group** | **n** | **Median** | **SD** | **Comparison** | ***P-*value** |
| --- | --- | --- | --- | --- | --- | --- |
| 6-KetoPGF1a | ntRag2 | 6 | 890.00 | 99.00 | ntRag2+FO-ntRag2 | 1 |
|  | ntRag2+FO | 6 | 920.00 | 580.00 | tRag2+FO-tRag2 | 0.19 |
|  | tRag2 | 10 | 550.00 | 310.00 | tRag2-ntRag2 | 0.093 |
|  | tRag2+FO | 10 | 400.00 | 220.00 | tRag2+FO-ntRag2+FO | 0.007 |
| TXB2 | ntRag2 | 6 | 76.00 | 14.00 | ntRag2+FO-ntRag2 | 0.18 |
|  | ntRag2+FO | 6 | 59.00 | 27.00 | tRag2+FO-tRag2 | 0.123 |
|  | tRag2 | 10 | 110.00 | 28.00 | tRag2-ntRag2 | 0.007 |
|  | tRag2+FO | 10 | 69.00 | 26.00 | tRag2+FO-ntRag2+FO | 0.155 |
| PGF2a | ntRag2 | 6 | 140.00 | 9.30 | ntRag2+FO-ntRag2 | 0.937 |
|  | ntRag2+FO | 6 | 160.00 | 120.00 | tRag2+FO-tRag2 | 0.393 |
|  | tRag2 | 10 | 97.00 | 29.00 | tRag2-ntRag2 | 0.22 |
|  | tRag2+FO | 10 | 81.00 | 55.00 | tRag2+FO-ntRag2+FO | 0.042 |
| 8-Iso | ntRag2 | 6 | 4.60 | 0.99 | ntRag2+FO-ntRag2 | 0.818 |
|  | ntRag2+FO | 6 | 5.60 | 4.30 | tRag2+FO-tRag2 | 0.197 |
|  | tRag2 | 10 | 3.90 | 0.68 | tRag2-ntRag2 | 0.353 |
|  | tRag2+FO | 10 | 3.20 | 1.80 | tRag2+FO-ntRag2+FO | 0.073 |
| PGE2 | ntRag2 | 6 | 480.00 | 95.00 | ntRag2+FO-ntRag2 | 1 |
|  | ntRag2+FO | 6 | 480.00 | 120.00 | tRag2+FO-tRag2 | 0.643 |
|  | tRag2 | 10 | 560.00 | 160.00 | tRag2-ntRag2 | 0.353 |
|  | tRag2+FO | 10 | 460.00 | 220.00 | tRag2+FO-ntRag2+FO | 0.958 |
| PGD2 | ntRag2 | 6 | 240.00 | 24.00 | ntRag2+FO-ntRag2 | 1 |
|  | ntRag2+FO | 6 | 230.00 | 180.00 | tRag2+FO-tRag2 | 0.579 |
|  | tRag2 | 10 | 250.00 | 120.00 | tRag2-ntRag2 | 0.814 |
|  | tRag2+FO | 10 | 230.00 | 140.00 | tRag2+FO-ntRag2+FO | 0.635 |
| 11DHy-TXB2 | ntRag2 | 6 | 0.14 | 0.19 | ntRag2+FO-ntRag2 | 0.394 |
|  | ntRag2+FO | 6 | 0.32 | 0.34 | tRag2+FO-tRag2 | 0.519 |
|  | tRag2 | 10 | 0.22 | 0.27 | tRag2-ntRag2 | 0.896 |
|  | tRag2+FO | 10 | 0.03 | 0.08 | tRag2+FO-ntRag2+FO | 0.088 |
| LXA4 | ntRag2 | 6 | 0.98 | 0.45 | ntRag2+FO-ntRag2 | 0.31 |
|  | ntRag2+FO | 6 | 0.59 | 0.69 | tRag2+FO-tRag2 | 0.353 |
|  | tRag2 | 10 | 2.10 | 1.50 | tRag2-ntRag2 | 1 |
|  | tRag2+FO | 10 | 0.90 | 0.89 | tRag2+FO-ntRag2+FO | 0.875 |
| LTB4 | ntRag2 | 6 | 6.40 | 2.40 | ntRag2+FO-ntRag2 | 0.331 |
|  | ntRag2+FO | 6 | 5.20 | 3.10 | tRag2+FO-tRag2 | 0.739 |
|  | tRag2 | 10 | 5.90 | 2.40 | tRag2-ntRag2 | 0.792 |
|  | tRag2+FO | 10 | 6.30 | 3.90 | tRag2+FO-ntRag2+FO | 0.382 |
| PGJ2 | ntRag2 | 6 | 7.50 | 0.76 | ntRag2+FO-ntRag2 | 0.18 |
|  | ntRag2+FO | 6 | 9.80 | 5.50 | tRag2+FO-tRag2 | 0.024 |
|  | tRag2 | 10 | 8.30 | 3.60 | tRag2-ntRag2 | 0.3 |
|  | tRag2+FO | 10 | 5.10 | 3.00 | tRag2+FO-ntRag2+FO | 0.042 |
| TRXA3 | ntRag2 | 6 | 9.20 | 4.50 | ntRag2+FO-ntRag2 | 0.093 |
|  | ntRag2+FO | 6 | 4.70 | 2.20 | tRag2+FO-tRag2 | 0.895 |
|  | tRag2 | 10 | 5.20 | 2.90 | tRag2-ntRag2 | 0.118 |
|  | tRag2+FO | 10 | 5.30 | 3.10 | tRag2+FO-ntRag2+FO | 0.98 |
| TRXB3 | ntRag2 | 6 | 12.00 | 9.90 | ntRag2+FO-ntRag2 | 0.065 |
|  | ntRag2+FO | 6 | 5.20 | 3.00 | tRag2+FO-tRag2 | 0.912 |
|  | tRag2 | 10 | 3.90 | 3.10 | tRag2-ntRag2 | 0.056 |
|  | tRag2+FO | 10 | 4.10 | 2.20 | tRag2+FO-ntRag2+FO | 0.635 |
| 5-HETE | ntRag2 | 6 | 8.10 | 3.90 | ntRag2+FO-ntRag2 | 0.31 |
|  | ntRag2+FO | 6 | 5.60 | 2.50 | tRag2+FO-tRag2 | 0.579 |
|  | tRag2 | 10 | 11.00 | 3.60 | tRag2-ntRag2 | 0.562 |
|  | tRag2+FO | 10 | 9.20 | 7.30 | tRag2+FO-ntRag2+FO | 0.263 |
| 8-HETE | ntRag2 | 6 | 5.40 | 2.10 | ntRag2+FO-ntRag2 | 0.093 |
|  | ntRag2+FO | 6 | 4.00 | 2.00 | tRag2+FO-tRag2 | 0.529 |
|  | tRag2 | 10 | 4.10 | 1.70 | tRag2-ntRag2 | 0.492 |
|  | tRag2+FO | 10 | 3.50 | 0.97 | tRag2+FO-ntRag2+FO | 0.958 |
| 9-HETE | ntRag2 | 6 | 9.50 | 1.80 | ntRag2+FO-ntRag2 | 0.485 |
|  | ntRag2+FO | 6 | 7.80 | 3.50 | tRag2+FO-tRag2 | 0.184 |
|  | tRag2 | 10 | 8.70 | 3.50 | tRag2-ntRag2 | 0.562 |
|  | tRag2+FO | 10 | 7.00 | 2.10 | tRag2+FO-ntRag2+FO | 0.275 |
| 11-HETE | ntRag2 | 6 | 80.00 | 8.30 | ntRag2+FO-ntRag2 | 1 |
|  | ntRag2+FO | 6 | 80.00 | 37.00 | tRag2+FO-tRag2 | 0.197 |
|  | tRag2 | 10 | 72.00 | 23.00 | tRag2-ntRag2 | 0.368 |
|  | tRag2+FO | 10 | 61.00 | 26.00 | tRag2+FO-ntRag2+FO | 0.147 |
| 12-HETE | ntRag2 | 6 | 98.00 | 33.00 | ntRag2+FO-ntRag2 | 0.132 |
|  | ntRag2+FO | 6 | 63.00 | 39.00 | tRag2+FO-tRag2 | 0.578 |
|  | tRag2 | 10 | 64.00 | 21.00 | tRag2-ntRag2 | 0.428 |
|  | tRag2+FO | 10 | 65.00 | 20.00 | tRag2+FO-ntRag2+FO | 0.854 |
| 15-HETE | ntRag2 | 6 | 110.00 | 17.00 | ntRag2+FO-ntRag2 | 0.851 |
|  | ntRag2+FO | 6 | 100.00 | 57.00 | tRag2+FO-tRag2 | 0.28 |
|  | tRag2 | 10 | 86.00 | 44.00 | tRag2-ntRag2 | 0.313 |
|  | tRag2+FO | 10 | 77.00 | 22.00 | tRag2+FO-ntRag2+FO | 0.093 |
| 5,6-EET | ntRag2 | 6 | 2.50 | 1.20 | ntRag2+FO-ntRag2 | 0.699 |
|  | ntRag2+FO | 6 | 2.10 | 1.20 | tRag2+FO-tRag2 | 0.009 |
|  | tRag2 | 10 | 2.90 | 1.10 | tRag2-ntRag2 | 0.562 |
|  | tRag2+FO | 10 | 1.60 | 0.77 | tRag2+FO-ntRag2+FO | 0.147 |
| 8,9-EET | ntRag2 | 6 | 0.71 | 0.46 | ntRag2+FO-ntRag2 | 0.18 |
|  | ntRag2+FO | 6 | 0.48 | 0.35 | tRag2+FO-tRag2 | 0.003 |
|  | tRag2 | 10 | 0.54 | 0.25 | tRag2-ntRag2 | 0.562 |
|  | tRag2+FO | 10 | 0.32 | 0.10 | tRag2+FO-ntRag2+FO | 0.147 |
| 11,12-EET | ntRag2 | 6 | 0.67 | 0.32 | ntRag2+FO-ntRag2 | 0.699 |
|  | ntRag2+FO | 6 | 0.56 | 0.35 | tRag2+FO-tRag2 | 0.052 |
|  | tRag2 | 10 | 0.92 | 0.73 | tRag2-ntRag2 | 0.313 |
|  | tRag2+FO | 10 | 0.37 | 0.37 | tRag2+FO-ntRag2+FO | 0.792 |
| 14,15-EET | ntRag2 | 6 | 0.47 | 0.17 | ntRag2+FO-ntRag2 | 0.485 |
|  | ntRag2+FO | 6 | 0.44 | 0.23 | tRag2+FO-tRag2 | 0.035 |
|  | tRag2 | 10 | 0.98 | 0.77 | tRag2-ntRag2 | 0.275 |
|  | tRag2+FO | 10 | 0.37 | 0.33 | tRag2+FO-ntRag2+FO | 0.635 |
