# Supplementary material for: Effects of increase in fish oil intake on intestinal eicosanoids and inflammation in a mouse model of colitis
Source: Lipids Health Dis. 2013 May 31;12:81. doi: 10.1186/1476-511X-12-81 (PMC3691874; doi:10.1186/1476-511X-12-81)
Supplement: Additional file 2 — List of DHA- and EPA-derived metabolites quantified in colon and values. Colon preparations of control and colitis animals under control- or FO-diet were analyzed. Medians are expressed in pg/mg of tissue. Significant differences between group comparisons are highlighted in grey. [file 1476-511X-12-81-S2.docx]

**Additional file 2:** List of DHA- and EPA-derived metabolites quantified in colon and values.

Colon preparations of control and colitis animals under control- or FO-diet were analyzed. Medians are expressed in pg/mg of tissue. Significant differences between group comparisons are highlighted in grey.

| **Metabolite (pg/mg)** | **Group** | **n** | **Median** | **SD** | **Comparison** | ***P-*value** |
| --- | --- | --- | --- | --- | --- | --- |
| D17-6keto-PGF1a | ntRag2 | 6 | 1.70 | 0.28 | ntRag2+FO-ntRag2 | 0.002 |
|  | ntRag2+FO | 6 | 13.00 | 6.20 | tRag2+FO-tRag2 | 0.009 |
|  | tRag2 | 10 | 1.30 | 0.72 | tRag2-ntRag2 | 0.22 |
|  | tRag2+FO | 10 | 3.50 | 3.60 | tRag2+FO-ntRag2+FO | 0.042 |
| TXB3 | ntRag2 | 6 | 0.78 | 0.23 | ntRag2+FO-ntRag2 | 0.002 |
|  | ntRag2+FO | 6 | 2.80 | 1.40 | tRag2+FO-tRag2 | <0.001 |
|  | tRag2 | 10 | 0.86 | 0.24 | tRag2-ntRag2 | 0.252 |
|  | tRag2+FO | 10 | 2.70 | 1.80 | tRag2+FO-ntRag2+FO | 0.875 |
| 8-Iso-PGF3a | ntRag2 | 6 | 2.80 | 0.26 | ntRag2+FO-ntRag2 | 1 |
|  | ntRag2+FO | 6 | 2.90 | 0.52 | tRag2+FO-tRag2 | 0.516 |
|  | tRag2 | 10 | 2.90 | 0.52 | tRag2-ntRag2 | 1 |
|  | tRag2+FO | 10 | 3.00 | 0.37 | tRag2+FO-ntRag2+FO | 0.476 |
| PGF3a | ntRag2 | 6 | 1.40 | 0.29 | ntRag2+FO-ntRag2 | 0.002 |
|  | ntRag2+FO | 6 | 4.20 | 0.95 | tRag2+FO-tRag2 | 0.002 |
|  | tRag2 | 10 | 1.60 | 0.23 | tRag2-ntRag2 | 0.155 |
|  | tRag2+FO | 10 | 2.90 | 1.80 | tRag2+FO-ntRag2+FO | 0.024 |
| PGE3 | ntRag2 | 6 | 3.50 | 0.67 | ntRag2+FO-ntRag2 | 0.002 |
|  | ntRag2+FO | 6 | 26.00 | 14.00 | tRag2+FO-tRag2 | <0.001 |
|  | tRag2 | 10 | 4.00 | 2.70 | tRag2-ntRag2 | 0.713 |
|  | tRag2+FO | 10 | 21.00 | 14.00 | tRag2+FO-ntRag2+FO | 0.635 |
| PGD3 | ntRag2 | 6 | 0.15 | 0.33 | ntRag2+FO-ntRag2 | 0.002 |
|  | ntRag2+FO | 6 | 4.20 | 3.00 | tRag2+FO-tRag2 | 0.006 |
|  | tRag2 | 10 | 0.06 | 0.09 | tRag2-ntRag2 | 0.944 |
|  | tRag2+FO | 10 | 1.50 | 1.80 | tRag2+FO-ntRag2+FO | 0.056 |
| 5-HEPE | ntRag2 | 6 | 0.25 | 0.38 | ntRag2+FO-ntRag2 | 0.041 |
|  | ntRag2+FO | 6 | 1.40 | 0.53 | tRag2+FO-tRag2 | <0.001 |
|  | tRag2 | 10 | 0.09 | 0.11 | tRag2-ntRag2 | 0.326 |
|  | tRag2+FO | 10 | 1.70 | 0.89 | tRag2+FO-ntRag2+FO | 0.875 |
| 8-HEPE | ntRag2 | 6 | 0.37 | 0.04 | ntRag2+FO-ntRag2 | 0.004 |
|  | ntRag2+FO | 6 | 0.76 | 0.21 | tRag2+FO-tRag2 | <0.001 |
|  | tRag2 | 10 | 0.30 | 0.03 | tRag2-ntRag2 | 0.042 |
|  | tRag2+FO | 10 | 0.66 | 0.20 | tRag2+FO-ntRag2+FO | 0.492 |
| 11-HEPE | ntRag2 | 6 | 1.80 | 0.60 | ntRag2+FO-ntRag2 | 0.002 |
|  | ntRag2+FO | 6 | 17.00 | 5.10 | tRag2+FO-tRag2 | <0.001 |
|  | tRag2 | 10 | 1.70 | 1.50 | tRag2-ntRag2 | 0.875 |
|  | tRag2+FO | 10 | 11.00 | 9.70 | tRag2+FO-ntRag2+FO | 0.056 |
| 12S-HEPE | ntRag2 | 6 | 2.70 | 1.30 | ntRag2+FO-ntRag2 | 0.002 |
|  | ntRag2+FO | 6 | 20.00 | 11.00 | tRag2+FO-tRag2 | <0.001 |
|  | tRag2 | 10 | 1.70 | 1.20 | tRag2-ntRag2 | 0.428 |
|  | tRag2+FO | 10 | 15.00 | 4.00 | tRag2+FO-ntRag2+FO | 0.171 |
| 15S-HEPE | ntRag2 | 6 | 2.20 | 1.70 | ntRag2+FO-ntRag2 | 0.002 |
|  | ntRag2+FO | 6 | 24.00 | 12.00 | tRag2+FO-tRag2 | <0.001 |
|  | tRag2 | 10 | 1.70 | 2.00 | tRag2-ntRag2 | 0.562 |
|  | tRag2+FO | 10 | 17.00 | 8.70 | tRag2+FO-ntRag2+FO | 0.252 |
| 17S-HDoHE | ntRag2 | 6 | 21.00 | 8.20 | ntRag2+FO-ntRag2 | 0.009 |
|  | ntRag2+FO | 6 | 55.00 | 29.00 | tRag2+FO-tRag2 | 0.015 |
|  | tRag2 | 10 | 20.00 | 14.00 | tRag2-ntRag2 | 0.875 |
|  | tRag2+FO | 10 | 51.00 | 22.00 | tRag2+FO-ntRag2+FO | 0.958 |
| 17R-Resolvin | ntRag2 | 6 | 1.10 | 0.02 | ntRag2+FO-ntRag2 | 1 |
|  | ntRag2+FO | 6 | 1.10 | 0.02 | tRag2+FO-tRag2 | 0.191 |
|  | tRag2 | 10 | 1.10 | 0.01 | tRag2-ntRag2 | 0.112 |
|  | tRag2+FO | 10 | 1.10 | 0.01 | tRag2+FO-ntRag2+FO | 0.573 |
| Resolvin-D1 | ntRag2 | 6 | 0.99 | 0.02 | ntRag2+FO-ntRag2 | 0.736 |
|  | ntRag2+FO | 6 | 0.99 | 0.03 | tRag2+FO-tRag2 | 0.399 |
|  | tRag2 | 10 | 0.99 | 0.01 | tRag2-ntRag2 | 0.226 |
|  | tRag2+FO | 10 | 0.99 | 0.01 | tRag2+FO-ntRag2+FO | 0.779 |
| Resolvin-D2 | ntRag2 | 6 | 0.00 | 0.00 | ntRag2+FO-ntRag2 | 0.455 |
|  | ntRag2+FO | 6 | 0.00 | 0.00 | tRag2+FO-tRag2 | 0.211 |
|  | tRag2 | 10 | 0.00 | 0.00 | tRag2-ntRag2 | 1 |
|  | tRag2+FO | 10 | 0.00 | 0.00 | tRag2+FO-ntRag2+FO | 1 |
| 10(S),17(S)-DiHDoHE | ntRag2 | 6 | 0.18 | 0.22 | ntRag2+FO-ntRag2 | 0.065 |
|  | ntRag2+FO | 6 | 0.68 | 0.33 | tRag2+FO-tRag2 | 0.009 |
|  | tRag2 | 10 | 0.19 | 0.30 | tRag2-ntRag2 | 0.983 |
|  | tRag2+FO | 10 | 0.75 | 0.47 | tRag2+FO-ntRag2+FO | 0.713 |
| LTB5 | ntRag2 | 6 | 0.45 | 0.01 | ntRag2+FO-ntRag2 | 0.024 |
|  | ntRag2+FO | 6 | 0.51 | 0.08 | tRag2+FO-tRag2 | <0.001 |
|  | tRag2 | 10 | 0.46 | 0.03 | tRag2-ntRag2 | 0.77 |
|  | tRag2+FO | 10 | 0.62 | 0.14 | tRag2+FO-ntRag2+FO | 0.147 |
| 8,9-EEP | ntRag2 | 6 | 0.61 | 0.07 | ntRag2+FO-ntRag2 | 0.937 |
|  | ntRag2+FO | 6 | 0.60 | 0.08 | tRag2+FO-tRag2 | 0.197 |
|  | tRag2 | 10 | 0.64 | 0.08 | tRag2-ntRag2 | 0.936 |
|  | tRag2+FO | 10 | 0.53 | 0.11 | tRag2+FO-ntRag2+FO | 0.635 |
| 17,18-EEP | ntRag2 | 6 | 1.60 | 0.33 | ntRag2+FO-ntRag2 | 0.002 |
|  | ntRag2+FO | 6 | 8.70 | 3.50 | tRag2+FO-tRag2 | <0.001 |
|  | tRag2 | 10 | 1.50 | 0.72 | tRag2-ntRag2 | 0.713 |
|  | tRag2+FO | 10 | 7.20 | 1.70 | tRag2+FO-ntRag2+FO | 0.087 |
