# Supplementary material for: Effects of increase in fish oil intake on intestinal eicosanoids and inflammation in a mouse model of colitis
Source: Lipids Health Dis. 2013 May 31;12:81. doi: 10.1186/1476-511X-12-81 (PMC3691874; doi:10.1186/1476-511X-12-81)
Supplement: Additional file 3 — Top 40 up-regulated colonic genes. Differentially expressed genes in colitis mice compared to control mice fed with control diet are shown. All with P < 0.001, with n = 8 and 9 mice for ntRag2 and tRag2 respectively. Illumina ID probe number is given. [file 1476-511X-12-81-S3.docx]

**Additional file 3:** Top 40 up-regulated colonic genes

Differentially expressed genes in colitis mice compared to control mice fed with control diet are shown. All with *P*<0.001, with n=8 and 9 mice for ntRag2 and tRag2 respectively. Illumina ID probe number is given.
